# Methods to measure quality of care and quality indicators through health facility surveys in low- and middle-income countries

**DOI:** 10.1093/intqhc/mzy136

**Published:** 2018-06-18

**Authors:** Diego Rios-Zertuche, Paola Zúñiga-Brenes, Erin Palmisano, Bernardo Hernández, Alexandra Schaefer, Casey K Johanns, Alvaro Gonzalez-Marmol, Ali H Mokdad, Emma Iriarte

**Affiliations:** 1 Salud Mesoamérica Initiative/Inter-American Development Bank, Calle 50, Edificio Tower Financial Center (Towerbank), Piso 23, Apartado postal: 0816-02900 zona 5, Panamá, Panamá; 2 Institute for Health Metrics and Evaluation, School of Medicine, University of Washington, 2301 Fifth Ave., Suite 600, Seattle, WA, USA

**Keywords:** quality of care, health facility surveys, quality metrics, clinical quality, performance measures, Central America, Salud Mesoamerica Initiative

## Abstract

**Objective:**

Present methods to measure standardized, replicable and comparable metrics to measure quality of medical care in low- and middle-income countries.

**Design:**

We constructed quality indicators for maternal, neonatal and child care. To minimize reviewer judgment, we transformed criteria from check-lists into data points and decisions into conditional algorithms. Distinct criteria were established for each facility level and type of care. Indicators were linked to discharge diagnoses. We designed electronic abstraction tools using computer-assisted personal interviewing software.

**Setting:**

We present results for data collected in the poorest areas of Belize, Costa Rica, El Salvador, Guatemala, Honduras, Nicaragua, Panama and the state of Chiapas in Mexico (January—October 2014).

**Results:**

We collected data from 12 662 medical records. Indicators show variations of quality of care between and within countries. Routine interventions, such as quality antenatal care (ANC), immediate neonatal care and postpartum contraception, had low levels of compliance. Records that complied with quality ANC ranged from 68.8% [confidence interval (CI):64.5–72.9] in Costa Rica to 5.7% [CI:4.0–8.0] in Guatemala. Less than 25% of obstetric and neonatal complications were managed according to standards in all countries.

**Conclusions:**

Our study underscores that, with adequate resources and technical expertise, collecting data for quality indicators at scale in low- and middle-income countries is possible. Our indicators offer a comparable, replicable and standardized framework to identify variations on quality of care. The indicators and methods described are highly transferable and could be used to measure quality of care in other countries.

## Introduction

Standardized, replicable and comparable metrics for quality of medical care in low- and middle-income countries are lacking [1, 2]. Poor quality is often attributed to lack of resources [1, 3, 4]; however, high variation in processes of care has been observed within countries and between countries [4]. Available data mostly considers aspects of healthcare infrastructure, availability of human resources, equipment and supplies, services provided, coverage and outcomes [5–8]. Quality perspectives from users and patients are also increasingly available [9, 10]. Yet, of the three categories described by Donabedian (structure, process and outcome) [11], a gap remains for the performance of processes of care [2]. Adequate healthcare is as much about process as it is about outcome [12, 13]. In most cases, the relation between processes and outcomes is not well understood [13]. Furthermore, outcome data is not useful to understand what processes need improvement [12].

In high-income countries, quality metrics are widely used and have become essential [14]. Data is regularly used to monitor healthcare quality, evaluate quality improvement efforts, implement pay-for-performance programs and reporting [15]. Unfortunately, these metrics often rely on sophisticated health information systems and electronic health records (EHR), which are still far from reality in many low- and middle-income countries [4, 16]. Even when data is available, the diversity in record-keeping practices and limited standardization create challenges to obtain comparable indicators [4, 15].

Medical records have been traditionally used for quality audits and improvement initiatives [12, 17]. This is not surprising; medical records are essential tools to evaluate the patient’s medical history and document their progress and care. As care is provided by a team of professionals over time, medical records allow for continuity of care during in- and out-patient encounters. Medical records also constitute legal documents that serve as evidence of care provided. Moreover, medical records have proven useful for quality improvement. Medical record audits, often combined with provider feedback, can improve compliance with clinical guidelines [16–18].

Different approaches have been used to measure quality from medical records, which can be broadly grouped in two categories: implicit review, which entails expert judgment and explicit review, which involves using previously defined criteria [19]. Each of these approaches has been perfected to improve inter-rater reliability, comparability and accuracy. Implicit reviews include structured methods to guide reviewers through each record [19]. On the contrary, explicit reviews evolved from procedure check-lists [20] to the abstraction of specific data [15, 19]—and the use of sophisticated methods, such as the use of search-terms and natural language processing programs, for EHRs [15]. Explicit methods are criticized mainly for over-simplification, while implicit methods are distrusted for poor inter-rater reliability [12, 18]. However, the correlation between both methods has been studied, concluding with moderately high convergence [19].

The use of explicit methods favors the creation of ‘quality indicators’ containing standards to evaluate clinical practice [21]. These indicators are developed using clinical guidelines and expert panels to select the most clinically significant measures [13, 22]. Quality indicators do not intend to become clinical guidelines, but to capture essential elements of processes of care [21]. Conditional logic and algorithms allow for indicators with increased complexity [12, 13]. Using this logic, it is possible to establish some criteria that are applicable to all patients, and others that can be restricted to patients with specific conditions [13]. Such algorithms have been commonly used to determine costs of care in diagnosis-related groups (DRGs). Patients are grouped into diagnoses categories and then evaluated to determine whether complications, comorbidities or other patient characteristics affect the use of hospital resources [23]. Although conditional logic and algorithms increase the complexity of data collection, computer-assisted data-abstraction software facilitates skip patterns, data quality checks and calculations during the abstraction process [24]. Likewise, statistical analysis software packages enable data processing and automation for indicator construction.

In this paper, we seek to answer: how to measure quality of care with standardized, replicable and comparable metrics? In particular, when EHRs are not available and recording practices are not consistent. First, we describe how quality indicators were constructed. Then, we explain the design of chart abstraction tools for the explicit medical record reviews. And finally, we illustrate the implementation of these methods through health facility surveys collected for ‘Salud Mesoamerica Initiative’ (SMI). Although our examples are based on indicators for maternal, neonatal and child care, we believe these methods can be applied to other processes of care. We hope to contribute to the foundation of urgently-needed metrics to measure quality of healthcare.

## Methods

### Indicator construction

We constructed quality indicators for maternal and child care (see Table 1). First, we used check-lists from a quality improvement initiative as an initial framework [20]. These check-lists helped us establish a reference for standards of care and provided us with actionable criteria for quality improvement. We compared the criteria against maternal and child health norms and protocols in each country. If check-lists for a desired process were not available, we reviewed clinical guidelines and consulted expert obstetricians and pediatricians from the region to select a subset of criteria for critical processes of care.

**Table 1:** Summary of quality indicators by life-cycle

Life-cycle	Quality indicators
Pregnancy	Antenatal care before 13 weeks gestation
	Quality antenatal care
Delivery	Use of partograph according to standards
	Oxytocin administration after birth
Complications	Obstetric complications managed according to standards
	Neonatal complications managed according to standards
Newborn	Immediate neonatal care with quality
Postpartum	Immediate postpartum care with quality
	Postpartum contraception
Children	Children who received two deworming doses
	Diarrhea in children treated with oral rehydration salts and zinc
	Follow-up for children with pneumonia within 2 days

Formulas and criteria for each indicator are included in Supplemental Annex A.

To minimize reviewer judgment, we transformed criteria from check-lists into data points and decisions into conditional algorithms. For example, instead of asking the reviewer if oxytocin was administered 1 min after birth, we asked them to record the time of birth, whether oxytocin was administered, and the time of administration of oxytocin. The algorithms were designed to be specific enough to measure compliance with clinical guidelines, but at the same time with built-in flexibility to allow variations in treatment due to physician preferences or patient conditions. For instance, obstetric hemorrhage following uterine atony could be managed using uterotonics, bimanual compression, uterine massage or other appropriate procedures.

**Figure 1:**
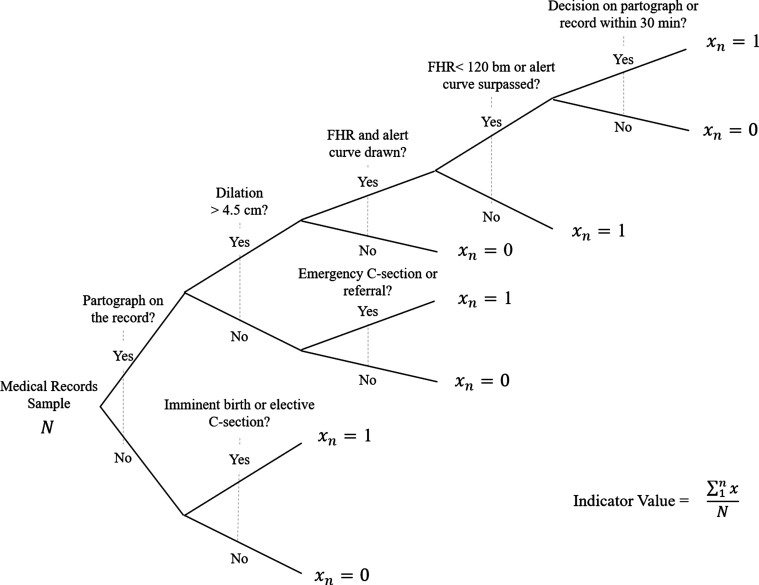
Sample algorithm for use of partograph according to standards indicator. Denominator: Total number of delivery records in the last 2 years in the sample. Numerator: Delivery records from Basic and Complete EONC: a partograph is included in the record and filled out completely (in cases where the woman did not arrive in imminent birth or for a C-section). If a partograph is completed and included in the record (regardless of the type of delivery) the following standards must be met: emergency C-section or referral (if dilation<4·5 cm) + Fetal heart rate and alert curves recorded (if dilation >4·5 cm) + a note is in the partograph/record within 30 min (if Fetal heart rate <120 bpm) + a note is in the partograph/record within 30 min (if alert curve is surpassed).

Moreover, we established distinct criteria for each level of care. Indicators were developed considering three levels of Essential Obstetric and Neonatal Care (EONC): ambulatory, encompassing outpatient care; basic, providing birth attention and basic emergency obstetric and neonatal care; and complete, facilities with an operating theater and health specialists. While some indicators applied only to ambulatory EONC, and others to basic and complete, in some cases the different capabilities of basic and complete facilities required separate treatment. That was the case for indicators of obstetric and neonatal complications, for which basic facilities were required to provide initial treatment and transfer the patient to complete facilities for full treatments. Yet, in some countries, a small number of basic facilities had some capabilities comparable to complete facilities (for example, an operating room and part-time availability of anesthesiologists). Considering such cases, the algorithms also provided flexibility for basic facilities to transfer patients or to provide full treatments. Most algorithms for routine care did not vary by level. A sample algorithm is shown in Fig. 1.

Indicators were linked to a set of discharge diagnoses, or encounter reasons, for the group of conditions measured. To comply with the indicator, the record under review had to meet all the required criteria. We selected the relevant diagnoses for each indicator using ICD-10 codes in hospitals, and discharge diagnoses or encounter descriptions in smaller facilities. For example, for indicators considering partograph use, we selected diagnoses of non-complicated deliveries and routine C-sections. For indicators considering obstetric complications, we selected the most common diagnoses for sepsis, hemorrhage, and severe pre-eclampsia and eclampsia. Discharge diagnoses warranted that processes being evaluated were aligned with conditions treated.

After algorithms for each indicator were designed, we reviewed it jointly with experts, obstetricians and pediatricians from Ministries of Health in each country. During field visits, we also analyzed information availability in medical records, reviewed record-keeping practices, and ensured that criteria were measurable. Formulas reported in this manuscript are not necessarily the same used for SMI’s pay-for-performance scheme.

### Electronic abstraction tools

We designed electronic abstraction tools using software for computer-assisted personal interviewing (DatStat Illume, Open Data Kit, and SurveyBe), which were installed in netbooks or tablets. Instruments included built-in quality controls, such as required responses, date checks (for instance, postnatal care could only occur after the delivery date), minimum and maximum parameters, and others. To avoid capturing personal identifying information, such as birth dates, the survey software rendered a deidentified database. We organized questionnaires by module for the group of diagnoses under review (normal deliveries, obstetric complications, neonatal complications, antenatal care (ANC), etc.). Multiple indicators could be collected from each module.

### Sample selection

The sample selection included a two-step process. First, we selected a random sample of health facilities serving the poorest areas of each country, stratified by EONC level. Then, we selected a sample of medical records from individual health facilities for target diagnoses within a predefined timeframe. If a random sample could not be selected using discharge diagnoses from the country’s information systems, a systematic sample of medical records was selected on-site. The systematic procedure encompassed estimating the number of cases for the target diagnosis in any given week, which would be the sampling interval, and selecting a random week as the starting point for medical record selection. Records for the target diagnosis would be included in the sample if they were directly selected or records two before or after the selected case. This procedure ensured the sample included records for the entire timeframe considered by the indicator. When the target sample size was equal to or smaller than the total number of cases available, all medical records were selected. The design allowed us to evaluate performance of the health system and that of individual facilities.

### Reviewer profiles

Most reviewers were medical doctors and nurses with 1–2 years of work experience. Reviewers were expected to collect all data individually for the less complex diagnoses, and in teams of two (one doctor and one nurse) for complications. In each country, teams of 4–8 reviewers were recruited. Field supervisors were also recruited to monitor quality and coordinate logistics.

### Training and pilot

Reviewer teams were trained in a 2-day workshop followed by a 2-day pilot. Training sessions included an overview of SMI, presentations on data collection procedures and confidentiality, walkthrough data-abstraction tools and practice sessions. Reviewer performance was closely monitored during all data collection, as data was regularly uploaded for analysis and quality checks.

### Data collection and analysis

We present results for data from Belize, Costa Rica, El Salvador, Guatemala, Honduras, Nicaragua, Panama and the state of Chiapas in Mexico (20 January 2014–24 October 2014). The survey methodology has been explained in detail [25]. Data collection was approved by institutional review boards at the University of Washington and data collection firms, as well as the Ministries of Health. No personally identifiable information was collected. Analyses were performed using Stata/SE 12.1 (StataCorp LP, College Station, TX). Although customized indicator criteria were developed per country, we used standardized formulas in our analyses for comparability (unless otherwise stated).

## Results

We collected data from 12 662 medical records in 8 countries (see Table 2). Indicators show variations of quality of care between countries (see Table 3). Routine interventions, such as quality ANC, immediate neonatal care, and postpartum contraception had low levels of compliance. For instance, less than one in every two newborns received all the required checkups except for Belize. Administration of oxytocin 1 min after birth was required by all country norms at the time of the survey except for Costa Rica. When oxytocin administration was required, countries met criteria for over 80% of the records. In comparison, immediate postpartum care with quality, including checkups within 2 h of birth, were only mandatory in Guatemala and Honduras. While around 40% of the records met the required criteria in Guatemala and Honduras, <15% of the records met the criteria in other countries. Less than 25% of obstetric and neonatal complications were managed according to standards in all countries.

**Table 2:** Health facility surveys sample description

Country	Health facilities	Medical records
Belize	38	1190
Costa Rica	60	1519
El Salvador	60	1591
Guatemala	60	2299
Honduras	60	1517
Chiapas, Mexico	60	1985
Nicaragua	60	1698
Panama	30	863

**Table 3:** Medical records from health facilities in the poorest areas of Mesoamerica that meet indicator criteria (January 2014–October 2014)

Indicator	Belize	Costa Rica	El Salvador	Guatemala	Honduras	Chiapas, Mexico	Nicaragua	Panama
	%	%	%	%	%	%	%	%[95% CI]
	[95% CI]	[95% CI]	[95% CI]	[95% CI]	[95% CI]	[95% CI]	[95% CI]	
Antenatal care before 13 weeks gestation	23	68.9	58.2	10.6	66.7	24.1	40.9	24.8
	[17.5–29.2]	[63.3–74.2]	[51–65.3]	[7.7–14.1]	[60.3–72.6]	[18.5–30.4]	[36.2–45.8]	[20.2–29.9]
Quality antenatal care	35.4	68.8	48	5.7	62.1	20	17.8	38.1
	[28.9–42.4]	[64.5–72.9]	[41.9–54.1]	[4–8]	[57.8–66.2]	[16.5–23.9]	[14.6–21.4]	[33.2–43.3]
Use of partograph according to standards	75.9	64.5		55.2	75.7	22.2	93.8	
	[65.3–84.6]	[54.9–73.4]		[48.9–61.3]	[64–85.2]	[12.7–34.5]	[88.2–97.3]	
Oxytocin administration after birth	80	32.6	89.9	94.4	94.5	83.6	84.5	93
	[70.2–87.7]	[24.7–41.3]	[84.7–93.8]	[91.2–96.7]	[90.7–97]	[79.2–87.4]	[79.2–88.8]	[89–95.9]
Obstetric complications managed according to standards^a^	13.6	16	12.8	6.5	6.8	21.8	13.7	
	[6–25]	[9.4–24.7]	[8.6–18.1]	[3.9–10]	[3.7–11.1]	[17.6–26.5]	[9.6–18.7]	
Neonatal complications managed according to standards^a^	7.5	1.8	7.5	1.2	1.5	3.8	1.7	
	[1.6–20.4]	[0.2–6.3]	[4.3–12.1]	[0.02–3.5]	[0.3–4.3]	[1.9–7]	[0.5–4.4]	
Immediate neonatal care with quality	86.8	41.7	38.3	33	47.6	39.6	53	24.5
	[76.4–93.8]	[33–50.8]	[29.1–48.2]	[27.6–38.8]	[38.5–56.7]	[34.1–45.3]	[45.5–60.3]	[19.2–30.4]
Immediate postpartum care with quality^1^	1.7	0	0	46.3	41.1	0.7	0	10.9
	[0–8.9]	[0–2.9]	[0–3.8]	[40.4–52.3]	[29.7–53.2]	[0.1–2.3]	[0–2.1]	[7.2–15.6]
Postpartum contraception^2^ (sterilization, oral contraceptives, implant, IUD and barrier methods)	1.5	7.9	2.7	1.4	24.8	10.1	16.9	3.3
	[0–8.2]	[3.9–14.1]	[0.6–7.7]	[0.4–3.5]	[19.4–30.8]	[7–13.9]	[11.7–23.9]	[1.4–6.4]
Children who received two deworming doses	14.2							
	[8.5–21.7]							
Diarrhea in children treated with oral rehydration salts and zinc	1.4		68.4		38.9	2.6		
	[0–7.4]		[61.7–74.6]		[32.5–45.6]	[0.8–5.9]		
Follow-up for children with pneumonia within 2 days					54.7			
					[44.8–64.4]			

^a^Country-specific formulas were used to calculate these indicators (see Supplemental Annex B).

Values show the percentage of medical records that meet criteria. 95% confidence intervals (CI) in brackets. Supplemental Annex A describes the formulas and criteria used to calculate each indicator. 1. At the time of the survey, immediate postpartum care, as defined by this indicator, was only part of country norms in Guatemala and Honduras. 2. Although injectable contraceptives postpartum (within 48 h of birth) are allowed by some country norms, they are not included in the definition considering the recommendations of the World Health Organization on the Medical eligibility criteria for contraceptive use (2015).

Table 4 shows percentage of medical records that meet each criterion of quality ANC. Only in Honduras and Costa Rica over 50% of records met the criteria for this indicator. Costa Rica and Belize have generally high coverage of ANC visits, and checkups are routinely performed on every visit; however, in Belize less than one of every two pregnant women received the required lab tests.

**Table 4:** Medical records from ambulatory health facilities in the poorest areas of each country that meet criteria for quality antenatal care (January 2014–October 2014)

	Belize	Costa Rica	El Salvador	Guatemala	Honduras	Chiapas, Mexico	Nicaragua	Panama
	%	%	%	%	%	%	%	%
	[95% CI]	[95% CI]	[95% CI]	[95% CI]	[95% CI]	[95% CI]	[95% CI]	[95% CI]
Observations	206	487	271	558	522	475	517	367
4+ ANC visits	92.2	77.2	66.8	22.8	67.8	67.8	30.2	58.3
	[87.7–95.5]	[73.2–80.9]	[60.8–72.4]	[19.3–26.5]	[63.6–71.8]	[63.4–72]	[26.2–34.3]	[53.1–63.4]
Doctor or nurse at each visit	86.9	77.2	66.8	12.5	67.8	66.3	29.4	53.4
	[81.5–91.2]	[73.2–80.9]	[60.8–72.4]	[9.9–15.6]	[63.6–71.8]	[61.9–70.6]	[25.5–33.5]	[48.2–58.6]
Vital signs checked at each visit	92.2	77.2	66.4	19.7	67.4	66.7	30.2	56.9
	[87.7–95.5]	[73.2–80.9]	[60.5–72]	[16.5–23.3]	[63.2–71.4]	[62.3–71]	[26.2–34.3]	[51.7–62.1]
Fundal height if gestational age >13 weeks	90.3	73.7	92.6	18.6	64.4	65.1	27.5	55.6
	[85.4–94]	[69.6–77.6]	[88.8–95.4]	[15.5–22.1]	[60.1–68.5]	[60.6–69.3]	[23.7–31.5]	[50.3–60.7]
Fetal checks if gestational age >20 weeks	88.8	72.3	97	17.9	65.9	57.5	28.6	55.3
	[83.7–92.8]	[68.1–76.2]	[94.3–98.7]	[14.8–21.4]	[61.7–70]	[52.9–62]	[24.8–32.7]	[50.1–60.5]
Lab tests performed at least once	40.3	92.8	69.7	48.4	87.7	26.1	73.7	56.9
	[33.5–47.3]	[90.1–94.9]	[63.9–75.2]	[44.2–52.6]	[84.6–90.4]	[22.2–30.3]	[69.7–77.4]	[51.7–62.1]
Quality ANC	35.4	68.8	48	5.7	62.1	20	17.8	38**.1**
	[28.9–42.4]	[64.5–72.9]	[41.9–54.1]	[4**–**8]	[57.8–66.2]	[16.5–23.9]	[14.6–21.4]	[33.2–43.3]

Values show the percentage of medical records that meet each criterion. To meet indicator requirements, all criteria required by the indicator had to be met by the medical record. 95% confidence intervals (CI) in brackets.

Table 5, shows the proportion of records meeting the criteria by each health facility in Chiapas, Mexico, for the application of oxytocin after birth. Although the average country score is 83.6% [95% confidence interval (CI): 79.2–87.4%], four health facilities scored much lower.

**Table 5:** Medical records from health facilities in the poorest areas of Chiapas, Mexico, that meet criteria for oxytocin administration after birth (January 2014–October 2014)

Health facility	EONC level	Observations	Oxytocin after birth % [95% CI]
Chiapas-50	Basic	17	47.1 [23–72.2]
Chiapas-65	Complete	40	47.5 [31.5–63.9]
Chiapas-53	Basic	6	50 [11.8–88.2]
Chiapas-58	Basic	12	58.3 [27.7–84.8]
Chiapas-77	Basic	17	88.2 [63.6–98.5]
Chiapas-35	Complete	50	90 [78.2–96.7]
Chiapas-49	Complete	50	92 [80.8–97.8]
Chiapas-38	Complete	46	93.5 [82.1–98.6]
Chiapas-42	Basic	16	93.8 [69.8–99.8]
Chiapas-43	Basic	23	95.7 [78.1–99.9]
Chiapas-76	Complete	25	96 [79.6–99.9]
Chiapas-57	Complete	34	100 [89.7–100]
Country score		336	83.6 [79.2–87.4]

Values show the percentage of medical records that meet criteria for each health facility in the poorest areas of Chiapas, Mexico. Health facilities classified by Essential Obstetric and Neonatal Care (EONC) levels. 95% confidence intervals (CI) in brackets.

## Discussion

Our study underscores that, with adequate resources and technical expertise, collecting data for quality indicators at scale in low- and middle-income countries is possible. Our indicators offer a comparable, replicable and standardized framework to identify variations on quality of care within and between countries. Our quality indicators and methods are highly transferable and could be used to measure quality of care in other countries. The proposed methods are also well-fitted for strategic decision-making and have important applications for operations planning and quality improvement.

Our methods to measure quality indicators through health facility surveys offer several advantages. First, our methods are rigorous and replicable. Anyone collecting data for the same indicators would obtain similar results (within the confidence interval), even if a different sample of records is selected—we tested this hypothesis in practice with consistent findings. Second, these methods are highly transferable and can be adapted. Although the learning curve is steep, our progress allows others to modify and implement these methods in different countries and contexts at a lower cost. The richness of the data collected has multiple potential applications—such as country-level comparisons, supporting strategic decision-making and quality improvement at multiple levels. Third, although we recommend that medical record reviews are performed by health professionals, recent graduates are usually well-fitted for the task, which reduces costs associated with data collection. Standardization is possible through short training sessions and frequent data quality checks. Fourth, designing and piloting data collection instruments itself can provide valuable recommendations to improve health systems. The systematic review of tools and processes reveals redundancies, duplication in recording, use of incorrect formats, and others. In one facility, we found the same ANC data recorded up to four times in different books, which was a burden on the facility’s staff. Fifth, additional criteria may be added to raise the bar for health facility performance.

From an operational perspective, a key advantage of our methods is the emphasis on uncovering process problems rather than individual errors. In implicit medical record review processes, the reviewers may be inclined to blame quality problems on specific health professionals. Our approach, on the contrary, focuses on processes and favors the analysis of aggregated data, instead of relying on the reviewer’s judgment. Eliciting process problems is an essential step to identify capability traps and implement quality improvement initiatives successfully [26]. Interestingly, in our example of quality ANC (Table 4), other than lab tests and qualified staff, most unmet criteria could be fulfilled with basic resources. Hence, our results underline the need to establish systematic processes of care and standardize healthcare delivery.

Moreover, these methods can also be used by ministries of health to monitor their own performance. Belize’s Ministry of Health is already performing regular measurements in health facilities. Similar methods are being implemented by quality improvement teams in several countries to monitor their own performance. Since data can be collected electronically on mobile-devices, and data processing and analysis can be automated into electronic dashboards, these methods can provide timely quality metrics for decision-making.

Our methods also had limitations. We found that not all data could be measured accurately within the medical record. Although information on the patient’s medical history, treatments and checkups was generally available, other data was hard to find—such as the physician’s area of specialization. We also could not measure how the procedures were performed or patient–physician interactions. Moreover, given that most records are paper-based and facilities are not linked to each other, we had trouble checking if users sought care in multiple health facilities, unless documentation was available on the record. Given that the sample considers people who received care in health facilities, these methods are not appropriate to measure coverage.

Medical records reviews have also been criticized for measuring documentation practices instead of quality of care. A recent study found that findings from medical record reviews obtained a score 10 percentage points lower than standardized patients [24]. Nevertheless, enforcing documentation practices would improve the accuracy of the data abstracted. In fact the patient’s progress is assessed in the medical record, such initiative would have a direct impact on quality. Further, it could prompt health practitioners to comply with clinical guidelines, which has led to improved outcomes [27, 28].

Moreover, we did not establish the relationship between quality indicators and outcomes. Further research is needed to understand this association. We were also not able to measure inter-rater reliability. Hence, we could not compare the reliability of our methods with others. From our empirical experience, inter-rater reliability decreases when data collected is complex and documentation practices are poor. Lastly, indicators’ criteria were selected for the countries under study; to be used globally, criteria may need revisions.

In fact, other methods have been used to collect data on quality of care [4, 29]. Unfortunately, all methods have limitations. Standardized patients are impractical to monitor quality regularly and present challenges evaluating processes for younger or older patients [4]. Exit interviews rely on the user’s understanding of the processes of care and the encounter’s outcome. Direct observation and recording visits create participation bias and standardizing observers is difficult [4]. Our methods are particularly useful for use at scale. Other methods would also be needed to gain in-depth insights of quality. As no method is immune to gaming [30], using multiple methods whenever possible is advised.

As countries continue progress towards universal healthcare coverage, advancing quality of health in the global health agenda should be prioritized. Measuring quality indicators in national health surveys, like the MICS [5] and the SARA [8], could be an initial step. We showed that measuring quality of care is possible even in challenging environments such as the poorest areas of Mesoamerica. Our success is grounded on a strong team composed of survey specialists and health experts who know the countries and health systems. Buy-in from Ministries of Health and support from partners in the region were also critical during the indicator review and data collection processes. SMI made a great investment in a public good that can be easily modified and applied. Our team is happy to help others translating and implementing these methods.

## Supplementary Material

Supplementary Data

Supplementary Data
