# Supplementary material for: Methods to measure quality of care and quality indicators through health facility surveys in low- and middle-income countries
Source: Int J Qual Health Care. 2018 Jun 18;31(3):183–90. doi: 10.1093/intqhc/mzy136 (PMC6464097; doi:10.1093/intqhc/mzy136)
Supplement: Supplementary Data [file mzy136_supplementalannexa_jun_16_17.docx]

**Supplemental Annex A. General quality of care indicator definitions, formulas and criteria**

Contents

[Antenatal care before 13 weeks gestation 2](#_Toc485405956)

[Quality antenatal care 2](#_Toc485405957)

[Delivery 2](#_Toc485405958)

[Use of partograph according to standards 2](#_Toc485405959)

[Oxytocin administration after birth 3](#_Toc485405960)

[Complications 3](#_Toc485405961)

[Obstetric complications managed according to standards 3](#_Toc485405962)

[Neonatal complications managed according to standards 5](#_Toc485405963)

[Newborn 7](#_Toc485405964)

[Immediate neonatal care with quality 7](#_Toc485405965)

[Postpartum 8](#_Toc485405966)

[Immediate postpartum care with quality 8](#_Toc485405967)

[Postpartum contraception 8](#_Toc485405968)

[Children 8](#_Toc485405969)

[Deworming in children 8](#_Toc485405970)

[Diarrhea in children treated with oral rehydration salts and zinc 9](#_Toc485405971)

[Follow-up for pneumonia within 2 days 9](#_Toc485405972)

**Pregnancy**

## Antenatal care before 13 weeks gestation

*Definition:* Proportion of women who received ANC within first trimester

*Denominator:*

Total number of antenatal care (ANC) records in the sample.

*Formula:*

*Records from Ambulatory and Basic EONC Health Facilities that provide ANC*: First ANC visit performed by doctor or nurse + (date of 1^st^ ANC visit – date of last menstrual period = before to 13 weeks)

## Quality antenatal care

*Definition:* Proportion of women who received at least 4 ANC visits according to standards in the last two years

*Denominator*:

Total number of records of women who had their most recent pregnancy in the last two years and attended the health facility for antenatal care (ANC) in the sample.

*Formula*:

*Antenatal Medical Records from Ambulatory and Basic EONC Health Facilities that provide ANC*: Observe the following in the record: woman had 4 ANC visits, each with the following: attention by doctor or nurse + checks performed (weight + blood pressure) + fundal height if gestational age is more than 13 weeks + fetal checks (fetal heart rate + fetal movement) if gestational age is more than 20 weeks + Lab tests performed at least once (blood type + Rh factor + blood glucose level + HIV test + hemoglobin level + urinalysis).

# Delivery

## Use of partograph according to standards

*Definition:* Partograph used according to standards for births in the last two years

*Denominator:*

Total number of delivery records in the last two years in the sample.

*Formula:*

*Normal Delivery Records from Basic and Complete EONC Health Facilities*: A partograph is included in the record and filled out completely (in cases where the woman did not arrive in imminent birth or for a C-section). If a partograph is completed and included in the record (regardless of the type of delivery) the following standards must be met: Emergency C-Section or referral (if dilation<4.5 cm) + Fetal heart rate & alert curves recorded (if dilation >4.5cm) + a note is in the partograph/record within 30 minutes (if Fetal heart rate < 120 bpm) + a note is in the partograph/records within 30 minutes (if alert curve is surpassed).

## Oxytocin administration after birth

*Definition:* Application of oxytocin or other uterotonics following birth for delivery records in the past two years

*Denominator:*

Total number of delivery records in the sample.

*Formula:*

*Basic or Complete*: Oxytocin or other uterotonic was administered after delivery

# Complications

## Obstetric complications managed according to standards

*Definition:* Women with obstetric complications (sepsis, hemorrhage, severe pre-eclampsia and eclampsia) managed according to the norm in the last two years

*ICD-10 Codes for sample selection:*

| **Diagnosis** | **ICD-10** |
| --- | --- |
| *Hemorrhage:* | |
| Delayed or excessive hemorrhage following incomplete spontaneous abortion | O03.1 |
| Delayed or excessive hemorrhage following complete or unspecified spontaneous abortion | O03.6 |
| Delayed or excessive hemorrhage following abortion and ectopic and molar pregnancy | O08.1 |
| Placenta previa with hemorrhage | O44.1 |
| Premature separation of placenta with coagulation defect | O45.0 |
| Premature separation of placenta, unspecified | O45.9 |
| Rupture of uterus during labor | O71.1 |
| Postpartum inversion of uterus | O71.2 |
| Third-stage hemorrhage | O72.0 |
| Other immediate postpartum hemorrhage | O72.1 |
| Delayed and secondary postpartum hemorrhage | O72.2 |
| *Severe Pre-Eclampsia / Eclampsia:* | |
| Severe pre-eclampsia | O14.1 |
| HELLP syndrome | O14.2 |
| Eclampsia in pregnancy | O15.0 |
| Eclampsia in labor | O15.1 |
| Eclampsia in the puerperium | O15.2 |
| Eclampsia, unspecified as to time period | O15.9 |
| *Sepsis:* | |
| Puerperal sepsis | O85 |

*Denominator*:

Total number of obstetric complication records in the sample.

*Formula*:

Sepsis:

*Sepsis Medical Records from Basic EONC Health Facilities*: Temperature + pulse + blood pressure + antibiotic administration + adequate treatment

Treatment options:

- If septic abortion: MVA or instrumental curettage or hysterectomy or transfer to Complete
- If uterine perforation: surgical repair or hysterectomy or transfer to Complete
- If pelvic abscess: laparotomy or drainage or hysterectomy or surgical repair or transfer to Complete
- If postpartum endometritis: antibiotic administration or transfer to Complete
- If retained product: instrumental curettage or laparotomy or hysterectomy or transfer to complete
- If puerperal fever: antibiotic administration or transfer to Complete

*Sepsis Medical Records from Complete EONC Health Facilities*: Temperature + pulse + blood pressure + complete blood count (hemoglobin + hematocrit + platelets + leukocytes) + antibiotic administration + adequate treatment

Treatment options:

• If septic abortion: MVA or instrumental curettage or hysterectomy

• If uterine perforation: surgical repair or hysterectomy

• If pelvic abscess: laparotomy or drainage or hysterectomy or surgical repair

• If postpartum endometritis: antibiotic administration

• If retained product: instrumental curettage or laparotomy or hysterectomy

• If puerperal fever: antibiotic administration

Hemorrhage:

*Hemorrhage Medical Records from Basic EONC Health Facilities*: Pulse + blood pressure + Ringer/Hartman lactate or saline solution + (adequate management or transfer to Complete).

Management options:

- If hemorrhage following incomplete or complete abortion: MVA or instrumental curettage or transfer to Complete
- If ectopic pregnancy: laparotomy or salpingectomy or surgical repair or transfer to Complete
- If placenta previa with hemorrhage: C-section or hysterectomy or transfer to Complete
- If uterine rupture: laparotomy or hysterectomy or surgical repair or C-section or transfer to Complete
- If uterine atony: uterotonics (oxytocin or others) + bimanual compression or uterine massage or hydrostatic balloon or uterine tamponade or hypogastric artery ligation or uterine artery ligation or B-lynch suture or transfer to Complete
- If uterine inversion: {uterotonics (oxytocin or others) + repositioning of the uterus with anesthesia or sedation (nonsurgical procedures or surgical procedures)} or hysterectomy or transfer to Complete
- If retained product: uterotonics (oxytocin or others) + manual extraction or instrumental curettage or transfer to Complete

*Hemorrhage Medical Records from Complete EONC Health Facilities*: Pulse + Hematocrit + Hemoglobin + platelet count + blood pressure + Ringer/Hartman lactate or saline solution + adequate management

Management options:

- If hemorrhage following incomplete or complete abortion: MVA or instrumental curettage
- If ectopic pregnancy: laparotomy or salpingectomy or surgical repair
- If placenta previa with hemorrhage: C-section or hysterectomy
- If uterine rupture: laparotomy or hysterectomy or surgical repair or C-section
- If uterine atony: uterotonics (oxytocin or others) or bimanual compression or uterine massage or hydrostatic balloon or uterine tamponade or hypogastric artery ligation or uterine artery ligation or B-lynch suture or hysterectomy
- If uterine inversion: uterotonics (oxytocin or others) + repositioning of the uterus with anesthesia or sedation (nonsurgical procedures or surgical procedures) or hysterectomy
- If retained product: uterotonics (oxytocin or others) + manual extraction or instrumental curettage

Severe Pre-Eclampsia / Eclampsia:

*Severe Pre-Eclampsia Medical Records from Basic EONC Health Facilities*: systolic BP + diastolic BP + check for urine protein + Ringer/Hartman lactate or saline solution + magnesium sulfate + transfer to Complete.

*Severe Pre-Eclampsia Medical Records from Complete EONC Health Facilities*: Observe the following in the record: systolic BP + diastolic BP + pulse + respiratory rate + patellar reflex + if diastolic BP>110: (hydralazine or labetalol or nifedipine) + magnesium sulfate + check for urine protein+ platelet count + (Aspartate aminotransferase or serum glutamic oxaloacetic transaminase) + (Alanine aminotransferase or serum glutamate-pyruvate transaminase) + if 24=< gestational age <34 weeks: (Dexamethasone or betamethasone).

## Neonatal complications managed according to standards

*Definition:* Neonates with complications (low birth weight, prematurity, birth asphyxia and sepsis) managed according to standards in the last two years

*ICD-10 Codes for sample selection:*

| **Diagnosis** | **ICD-10** |
| --- | --- |
| *Low Birth Weight:* | |
| Extremely low birth weight | P07.0 |
| Other low birth weight | P07.1 |
| *Preterm:* | |
| Extreme immaturity | P07.2 |
| Other preterm infants | P07.3 |
| *Asphyxia:* | |
| Severe birth asphyxia | P21.0 |
| Mild and moderate birth asphyxia | P21.1 |
| Birth asphyxia, unspecified | P21.9 |
| *Sepsis:* | |
| Sepsis of newborn due to streptococcus, group B | P36.0 |
| Sepsis of newborn due to other and unspecified streptococci | P36.1 |
| Sepsis of newborn due to Staphylococcus aureus | P36.2 |
| Sepsis of newborn due to other and unspecified staphylococci | P36.3 |
| Sepsis of newborn due to Escherichia coli | P36.4 |
| Sepsis of newborn due to anaerobes | P36.5 |
| Other bacterial sepsis of newborn | P36.8 |
| Bacterial sepsis of newborn, unspecified | P36.9 |

*Denominator*:

Total number of records of neonates with birth complications (low birth weight, prematurity, birth asphyxia, or sepsis) in the sample.

*Formula*:

Low birth weight:

*Low Birth Weight Medical Records from Basic EONC Health Facilities*: Weight < 2500mg: Evaluated by doctor + Gestational age (Capurro or Ballard) + Weight + [if the baby was born in the same facility: classification according to weight (Low weight<2500 gr or extreme low weight < 1500 gr)] + Heart rate + Respiratory rate + Length + Head circumference + Skin color + Early breastfeeding or any glucose solution (oral or IV) + Warm Chain (warm sheets or radiant warmer or incubator) + if no complications are present: (observation or Transfer to Complete) + if complications are present {(respiratory: pneumonia or respiratory distress) or (neurologic: convulsions) or digestive(diarrhea) or metabolic(hypoglycemia if glucose< 25mg/dl)} or weight less than 1500gr: Transfer to Complete.

*Low Birth Weight Medical Records from Complete EONC Health Facilities*: Weight < 2500mg: Evaluated by specialist or doctor + Gestational age + Weight + [if the baby was born in the same facility: classification according to weight (Low weight<2500 gr or extreme low weight < 1500 gr)] + Heart rate + Respiratory rate + Length + Head circumference + Skin color + Early breastfeeding or any glucose solution (oral or IV) + Warm Chain (warm sheets or radiant warmer or incubator) + (if pneumonia: antibiotics) or (if diarrhea: IV solution + antibiotics) or (if convulsions: anticonvulsive) or (if hypoglycemia if glucose< 25mg/dl: glucose IV).

Prematurity:

*Prematurity Medical Records from Basic EONC Health Facilities*: Gestational age < 37 weeks: Evaluated by doctor + Gestational age (Capurro or Ballard) + [if the baby was born in the same facility: Classification of newborn according to gestational age (small, large or adequate)] + Weight + Heart rate + Respiratory rate + Glycemia + Head circumference + Skin color + Warm Chain (warm sheets or radiant warmer or incubator) + Breastfeeding or any glucose solution (oral or IV) + if no complications are present: (observation or Transfer to Complete) + if complications are present {(respiratory: pneumonia or respiratory distress) or (neurologic: convulsions) o digestive(diarrhea) or metabolic(hypoglycemia if glucose< 25mg/dl } or less than or equal to 34 weeks of gestation: Transfer to Complete}.

*Prematurity Medical Records from Complete EONC Health Facilities*: Gestational age < 37 weeks: Evaluated by specialist or doctor + Gestational age (Capurro or Ballard) + [if the baby was born in the same facility: Classification of newborn according to gestational age (small, large or adequate)] + Weight + Heart rate + Respiratory rate + Glycemia + Oxygen saturation + Head circumference + Skin color + Warm Chain (warm sheets or radiant warmer or incubator) + Breastfeeding or any glucose solution (oral or IV) + (if pneumonia: antibiotics) or (if diarrhea: IV solution + antibiotics) or (if convulsions: anticonvulsive) or (if hypoglycemia if glucose< 25mg/dl: glucose IV).

Asphyxia:

*Asphyxia Medical Records from Basic EONC Health Facilities* [Only if birth occurred in the health facility]: Evaluated by doctor + Warm Chain (warm sheets or radiant warmer or incubator) + Heart rate + Respiratory rate + APGAR score at 1 minute + APGAR score at 5 minutes + if APGAR score at 5 minutes ≤3 [Oxygen (mask or head box or cone or hood or nasal cannula or mechanic ventilation or oxygen tank) + Ambu (Positive pressure ventilation) + Transfer to Complete (unless child died)].

*Asphyxia Medical Records from Complete EONC Health Facilities* [Only if birth occurred in the health facility]: valuated by specialist or doctor + Warm Chain (warm sheets or radiant warmer or incubator) + Heart rate + Respiratory rate + APGAR score at 1 minute + APGAR score at 5 minutes + if APGAR score at 5 minutes ≤3 [Oxygen (mask or head box or cone or hood or nasal cannula or mechanic ventilation or oxygen tank) + Ambu (Positive pressure ventilation) or endotraqueal intubation or chest compressions + Oxygen saturation].

Sepsis:

*Sepsis Medical Records from Basic EONC Health Facilities*: Evaluated by doctor + Temperature + Heart rate + Respiratory rate + Antibiotic + (if no pediatrician present: transfer to Complete) + (if pediatrician present: admission or [if hemodynamic failure or shock: transfer to Complete]).

*Sepsis Medical Records from Complete EONC Health Facilities*: Evaluated by specialist or doctor + Temperature + Heart rate + Respiratory rate + Oxygen saturation + Complete blood count (platelets + leukocytes + neutrophil count + hemoglobin + hematocrit) + Antibiotic + Protein C-Reactive + Abdominal exam.

# Newborn

## Immediate neonatal care with quality

*Definition:* Neonates who received care according to standards from medical personnel after birth in the last 2 years.

*Denominator:*

Total number of postpartum care records in the sample.

*Formula:*

*Medical Records from Basic and Complete EONC Health Facilities*: All procedures and checkups recorded (Apgar score at 1 and 5 minutes + head circumference + height + weight + oxytetracycline ophthalmic (such as prophylaxis or chloramphenicol) administration + pulse + respiratory rate + skin color + vitamin k administration)

# Postpartum

## Immediate postpartum care with quality

*Definition:* Institutional postpartum patients of reproductive age, evaluated and registered in clinical records, at least every 15 min during the first hour and 30 min until complete 2 hours, and when leaving hospital in the last two years

*Denominator*:

Total number of postpartum care records in the sample.

*Formula*:

*Medical Records from Basic and Complete EONC Health Facilities*: Observe the following in the record: following checks performed 4 times in the first hour after birth: diastolic and systolic blood pressure + temperature + pulse. Following checks performed 2 times in the second hour after birth: diastolic and systolic blood pressure + temperature + pulse. Following check performed at discharge: diastolic and systolic blood pressure + temperature + pulse.

## Postpartum contraception

*Definition:* Women of reproductive age (15-49 years old) who had an in-facility delivery and received postpartum contraception (oral contraceptive pill, implant, condom, IUD, or tubal ligation) in the last two years.

*Denominator:*

Total number of birth records in our sample.

*Formula:*

*Number of birth records in which women receive postpartum contraception (Oral contraceptive pill, implant condom, IUD, or tubal ligation).*

# Children

## Deworming in children

*Definition:* Children (12-59 months) who received two doses of de-worming treatment in the last year:

*Denominator:*

Total number of deworming records in the sample.

*Formula:*

*Deworming medical record*: Two doses of albendazole or mebendazole were prescribed.

## Diarrhea in children treated with oral rehydration salts and zinc

*Definition:* Number of diarrhea cases in children 0-59 months that received treated or were prescribed with oral rehydration solution (ORS) or intravenous fluids, and zinc in the last two years.

*Denominator:*

Total number of diarrhea cases in children 0-59 months in the last two weeks in the sample.

*Formula:*

*Diarrhea medical records*: Treatment or prescription of ORS or IV solution, and zinc was recorded

## Follow-up for pneumonia within 2 days

*Definition:* Children 0-59 months diagnosed with pneumonia and attended follow up appointment after two days

*Denominator*:

Total number of pneumonia records among children aged 0 to 59 months in the sample.

*Formula*:

*Ambulatory*: Observe the following in the record: date of the follow-up appointment for the child with pneumonia =< 2 days after the initial appointment or =< 3 days if initial appointment was on Friday.
