# Supplementary material for: Methods to measure quality of care and quality indicators through health facility surveys in low- and middle-income countries
Source: Int J Qual Health Care. 2018 Jun 18;31(3):183–90. doi: 10.1093/intqhc/mzy136 (PMC6464097; doi:10.1093/intqhc/mzy136)
Supplement: Supplementary Data [file mzy136_supplementalannexb_jun_16_17.docx]

**Supplemental Annex B. Country-specific formulas for obstetric and neonatal complications indicators**

**Contents**

[Belize 2](#_Toc485407557)

[Belize: Obstetric complications managed according to standards 2](#_Toc485407558)

[Belize: Neonatal complications managed according to standards 2](#_Toc485407559)

[Costa Rica 4](#_Toc485407560)

[Costa Rica: Obstetric complications managed according to standards 4](#_Toc485407561)

[Costa Rica: Neonatal complications managed according to standards 5](#_Toc485407562)

[El Salvador 8](#_Toc485407563)

[El Salvador: Obstetric complications managed according to standards 8](#_Toc485407564)

[El Salvador: Neonatal complications managed according to standards 8](#_Toc485407565)

[Guatemala 9](#_Toc485407566)

[Guatemala: Obstetric complications managed according to standards 9](#_Toc485407567)

[Guatemala: Neonatal complications managed according to standards 9](#_Toc485407568)

[Honduras 11](#_Toc485407569)

[Honduras: Obstetric complications managed according to standards 11](#_Toc485407570)

[Honduras: Neonatal complications managed according to standards 12](#_Toc485407571)

[Chiapas, Mexico 13](#_Toc485407572)

[Chiapas: Obstetric complications managed according to standards 13](#_Toc485407573)

[Chiapas: Neonatal complications managed according to standards 14](#_Toc485407574)

# Belize

## Belize: Obstetric complications managed according to standards

*Definition:* Women with obstetric complications (sepsis, hemorrhage, severe pre-eclampsia and eclampsia) managed according to the norm in the last two years

*Denominator*:

Total number of obstetric complication records in the sample.

*Formula*:

Sepsis:

*Sepsis Medical Records from Basic EONC Health Facilities*: temperature + pulse + systolic blood pressure + diastolic blood pressure + antibiotic administration.

*Sepsis Medical Records from Complete EONC Health Facilities*: temperature + pulse + systolic blood pressure + diastolic blood pressure + antibiotic administration.

Hemorrhage:

*Hemorrhage Medical Records from Basic EONC Health Facilities*: Observe the following in the record: vital signs checked (systolic blood pressure + diastolic blood pressure + pulse + respiratory rate) + lab tests performed (urine protein) + medication was administered (magnesium sulfate + hydralazine/nifedipine (if diastolic blood pressure is >110 bpm)).

*Hemorrhage Medical Records from Complete EONC Health Facilities*: Observe the following in the record: vital signs check (systolic blood pressure + diastolic blood pressure + pulse + respiratory rate + patellar reflex) + lab tests performed (urine protein + platelet count + aspartate aminotransferase + alanine aminotransferase + lactate dehydrogenase) + medication was administered (magnesium sulfate + hydralazine/nifedipine (if diastolic blood pressure is >110 bpm) + dexamethasone/betamethasone (if gestational age is 26-34 weeks)) + outcome of pregnancy (c-section/vaginal delivery/other).

Severe Pre-Eclampsia / Eclampsia:

*Severe Pre-Eclampsia or Eclampsia Medical Records from Basic EONC Health Facilities*: vital signs checked (systolic blood pressure + diastolic blood pressure + pulse + respiratory rate) + lab tests performed (urine protein) + medication was administered (magnesium sulfate + hydralazine/nifedipine (if diastolic blood pressure is >110 bpm)).

*Severe Pre-Eclampsia or Eclampsia Medical Records from Complete EONC Health Facilities*: Observe the following in the record: vital signs check (systolic blood pressure + diastolic blood pressure + pulse + respiratory rate + patellar reflex) + lab tests performed (urine protein + platelet count + aspartate aminotransferase + alanine aminotransferase + lactate dehydrogenase) + medication was administered (magnesium sulfate + hydralazine/nifedipine (if diastolic blood pressure is >110 bpm) + dexamethasone/betamethasone (if gestational age is 26-34 weeks)) + outcome of pregnancy (c-section/vaginal delivery/other).

## Belize: Neonatal complications managed according to standards

*Definition:* Neonates with complications (low birth weight, prematurity, birth asphyxia and sepsis) managed according to standards in the last two years

*Denominator*:

Total number of records of neonates with birth complications (low birth weight, prematurity, birth asphyxia, or sepsis) in the sample.

*Formula*:

Low birth weight:

*Low Birth Weight Medical Records from Basic EONC Health Facilities*: Observe the following in the record: pulse + respiratory rate + oxygen saturation + Silverman score + blood glucose level + baby was evaluated by a doctor at admission

*Low Birth Weight Medical Records from Complete EONC Health Facilities*: Observe the following in the record: pulse + respiratory rate + oxygen saturation + Silverman score + blood glucose level + baby was evaluated by a doctor at admission

Prematurity:

*Prematurity Medical Records from Basic EONC Health Facilities*: Observe the following in the record: pulse + respiratory rate + oxygen saturation + Silverman score + blood glucose level + baby was evaluated by a doctor at admission.

*Prematurity Medical Records from Complete EONC Health Facilities*: Observe the following in the record: pulse + respiratory rate + oxygen saturation + Silverman score + blood glucose level + baby was evaluated by a doctor at admission.

Asphyxia:

*Asphyxia Medical Records from Basic EONC Health Facilities*: Observe the following in the record: temperature + pulse + respiratory rate + blood glucose level + antibiotic treatment (ampicillin/gentamicin/other antibiotic) + Apgar score in 1 minute + Apgar score in 5 minutes + Hb + baby was evaluated by a doctor at admission.

*Asphyxia Medical Records from Complete EONC Health Facilities*: Observe the following in the record: temperature + pulse + respiratory rate + blood glucose level + antibiotic treatment (ampicillin/gentamicin/other antibiotic) + Silverman score + oxygen saturation + c-reactive protein + Hb + erythrocyte sedimentation rate + chest radiograph + baby was evaluated by a doctor at admission.

Sepsis:

*Sepsis Medical Records from Basic EONC Health Facilities*: Observe the following in the record: temperature + pulse + leukocyte count + baby was evaluated by a doctor at admission + treatment with antibiotics (ampicillin/gentamicin/other antibiotic).

*Sepsis Medical Records from Complete EONC Health Facilities*: Observe the following in the record: temperature + pulse + leukocyte count + c-reactive protein + erythrocyte sedimentation rate + baby was evaluated by a doctor at admission + treatment with antibiotics (ampicillin/gentamicin/other antibiotic).

# Costa Rica

## Costa Rica: Obstetric complications managed according to standards

*Definition:* Women with obstetric complications (sepsis, hemorrhage, severe pre-eclampsia and eclampsia) managed according to the norm in the last two years

*Denominator*:

Total number of obstetric complication records in the sample.

*Formula*:

Sepsis:

*Sepsis Medical Records from Basic EONC Health Facilities*: Observe the following in the record: vital signs checked (diastolic blood pressure + systolic blood pressure + temperature + pulse) + complete blood count/leukocyte count + antibiotics administered (amikacin/clindamycin/gentamicin/ampicillin/metronidazole/other antibiotic). The appropriate actions must also be taken, all scenarios are listed below:

- If the cause of sepsis is a septic abortion: MVA/instrumental curettage/woman was transferred to another facility
- If the cause of sepsis is a uterine perforation: hysterectomy/woman was transferred to another facility
- If the cause of sepsis is pelvic abscess: laparotomy/woman was transferred to another facility

*Sepsis Medical Records from Complete EONC Health Facilities*: Observe the following in the record: vital signs checked (diastolic blood pressure + systolic blood pressure + temperature + pulse) + complete blood count + platelet count + antibiotics administered (amikacin/clindamycin/gentamicin/ampicillin/metronidazole/other antibiotic). The appropriate actions must also be taken, all scenarios are listed below:

- If the cause of sepsis is a septic abortion: MVA/instrumental curettage
- If the cause of sepsis is a uterine perforation: hysterectomy
- If the cause of sepsis is pelvic abscess: laparotomy

Hemorrhage:

*Hemorrhage Medical Records from Basic EONC Health Facilities*: Observe the following in the record: vital signs checked (pulse + diastolic blood pressure + systolic blood pressure) + medication administered (oxytocin/other uterotonic (misoprostol, methylergometrine, etc.) + Ringer’s lactate/Hartmann’s solution). The appropriate actions must also be taken, all scenarios are listed below:

- *During the 1st half of pregnancy*:
  - - - If the cause of the hemorrhage is a complicated abortion: MVA/ instrumental curettage/woman was transferred to another facility
      - If the cause of the hemorrhage is ectopic pregnancy: laparotomy/woman was transferred to another facility
- *During the 2nd half of pregnancy*:
  - - - If the cause of the hemorrhage is placenta previa or placental abruption: vaginal birth/cesarean section/woman was transferred to another facility
- *At any point during the pregnancy:*
  - - - If the cause of the hemorrhage is uterine rupture: laparotomy/cesarean section/woman was transferred to another facility
- *Postpartum hemorrhage:*
  - - - If the cause of the hemorrhage is uterine atony: uterine packing/hysterectomy/woman was transferred to another facility
      - If the cause of the hemorrhage is cervical lacerations or vaginal tearing or vulva tearing: surgical repair/woman was transferred to another facility
      - If the cause of the hemorrhage is uterine inversion: resetting the uterus under general anesthesia with no surgical techniques (Johnson Maneuver)/resetting the uterus under general anesthesia with surgical techniques (Huntington or Haultaim Maneuvers/woman was transferred to another facility

*Hemorrhage Medical Records from Complete EONC Health Facilities*: Observe the following in the record: vital signs checked (pulse + diastolic blood pressure + systolic blood pressure) + oxytocin/other uterotonic administered if the woman was NOT referred from a basic facility (misoprostol, methylergometrine, etc.) + Ringer’s lactate/Hartmann’s solution + lab tests performed (hematocrit + hb + pt + ptt + platelet count). The appropriate actions must also be taken, all scenarios are listed below:

- *During the 1st half of pregnancy*:
  - - - If the cause of the hemorrhage is a complicated abortion: MVA/ instrumental curettage
      - If the cause of the hemorrhage is ectopic pregnancy: laparotomy
- *During the 2nd half of pregnancy*:
  - - - If the cause of the hemorrhage is placenta previa or placental abruption: vaginal birth/cesarean section
- *At any point during the pregnancy:*
  - - - If the cause of the hemorrhage is uterine rupture: laparotomy/cesarean section
- *Postpartum hemorrhage:*
  - - - If the cause of the hemorrhage is uterine atony: uterine packing/hysterectomy/oxytocin or other uterotonic administered
      - If the cause of the hemorrhage is cervical lacerations or vaginal tearing or vulva tearing: surgical repair
      - If the cause of the hemorrhage is uterine inversion: resetting the uterus under general anesthesia with no surgical techniques (Johnson Maneuver)/resetting the uterus under general anesthesia with surgical techniques (Huntington or Haultaim Maneuvers

Severe Pre-Eclampsia / Eclampsia:

*Severe Pre-Eclampsia or Eclampsia Medical Records from Basic EONC Health Facilities*: Observe the following in the record: vital signs checked (diastolic blood pressure + systolic blood pressure) + urine protein test + hydralazine/labetalol/nifedipine administered (if diastolic blood pressure > 110) + magnesium sulfate administered.

*Severe Pre-Eclampsia or Eclampsia Medical Records from Complete EONC Health Facilities*: vital signs checked (diastolic blood pressure + systolic blood pressure + pulse + respiratory rate + patellar reflex) + hydralazine/labetalol/nifedipine administered (if diastolic blood pressure > 110) + magnesium sulfate administered + dexamethasone/betamethasone administered (if gestational age is between 24-35 weeks) + laboratory tests performed (urine protein + platelet count + creatinine test + uric acid test + aspartate aminotransferase/glutamic-oxaloacetic transaminase (SGOT or GOT) + alanine aminotransferase + glutamic-pyruvic transaminase (SGPT or GPT) + lactate dehydrogenase (LDH)).

## Costa Rica: Neonatal complications managed according to standards

*Definition:* Neonates with complications (low birth weight, prematurity, birth asphyxia and sepsis) managed according to standards in the last two years

*Denominator*:

Total number of records of neonates with birth complications (low birth weight, prematurity, birth asphyxia, or sepsis) in the sample.

*Formula*:

Low birth weight:

*Low Birth Weight Medical Records from Basic EONC Health Facilities*: Observe the following in the record: neonate was evaluated by a doctor + gestational age + vital signs checked (weight + weight classification + heart rate + respiratory rate + Silverman-Anderson/Downes score + height + head circumference + skin evaluation/APGAR score) + neonate was fed (early feeding/breastfeeding/liquid glucose (oral or IV)) + neonate was transferred to another facility if it had any of the following:

- Neonate was < 1500gr
- Respiratory complications (pneumonia)
- Digestive complications (diarrhea)
- Neurological complications (convulsions, lethargic, not nursing)
- Metabolic complications

*Low Birth Weight Medical Records from Complete EONC Health Facilities*: Observe the following in the record: neonate was evaluated by a specialist + gestational age + vital signs checked (weight + weight classification + heart rate + respiratory rate + Silverman-Anderson/Downes score + height + head circumference + skin evaluation) + neonate was fed (early feeding/breastfeeding/liquid glucose (oral or IV)). The appropriate actions must also be taken, all scenarios are listed below:

- - If neonate has pneumonia: antibiotics
  - If neonate has diarrhea: liquids (maternal milk or oral rehydration salts) by oral or intravenous routes
  - If convulsions: anticonvulsants
  - If neonatal hypoglycemia: glucose IV

Prematurity:

*Prematurity Medical Records from Basic EONC Health Facilities*: Observe the following in the record: neonate was evaluated by a doctor + method used to calculate gestational age (Ballard score / Capurro test) + gestational age classification (small/large/suitable for EG) + vital signs checked (weight + heart rate + respiratory rate + Silverman-Anderson/Downes score + head circumference + skin color/APGAR score) + glucose (reactive or test strip) performed + heat was applied to neonate + neonate was fed (breastfeeding/glucose (oral glucose serum/IV glucose serum)/IV feeding) + neonate was fed (glucose serum/Hartmann’s solution/Ringer’s lactate/Saline solution) with IV fluids if gestational age < 34 weeks) + neonate was transferred to another facility.

*Prematurity Medical Records from Complete EONC Health Facilities*: Observe the following in the record: neonate was evaluated by a specialist + method used to calculate gestational age (Ballard score / Capurro test) + gestational age classification (small/large/suitable for EG) + vital signs checked (weight + heart rate + respiratory rate + Silverman-Anderson/Downes score + head circumference + skin color/APGAR score) + lab tests performed (glucose (reactive or test strip) + oxygen saturation) + neonate stayed in an incubator + neonate was fed (breastfeeding/glucose (oral glucose serum/IV glucose serum)/IV feeding) + neonate was fed (glucose serum/Hartmann’s solution/Ringer’s lactate/Saline solution) with IV fluids if gestational age < 34 weeks).

Asphyxia:

*Asphyxia Medical Records from Basic EONC Health Facilities*: Observe the following in the record: neonate was evaluated by a doctor + the listed procedures were performed on the neonate (dried off + stimulated + positive pressure ventilation (AMBU) + applied heat) + vital signs checked (APGAR score is 0-7 at 1 minute + APGAR score at 5 minutes + heart rate + respiratory rate + skin color) + neonate is transferred to a complete facility if APGAR score > 7 at 5 minutes.

*Asphyxia Medical Records from Complete EONC Health Facilities*: Observe the following in the record: neonate was evaluated by a specialist + the listed procedures were performed on the neonate (dried off + stimulated + positive pressure ventilation (AMBU) + applied heat + oxygen application) + vital signs checked (APGAR score is 0-7 at 1 minute + APGAR score at 5 minutes + heart rate + respiratory rate + skin color).

Sepsis:

*Sepsis Medical Records from Basic EONC Health Facilities*: Observe the following in the record: neonate was evaluated by a doctor + vital signs checked (pulse + temperature + heart rate + respiratory rate + abdominal examination) + antibiotics administered (ampicillin/gentamicin/other antibiotic) + lab tests performed (complete blood count (leukocytes, hemoglobin and hematocrit) + platelet count). The appropriate actions must also be taken, all scenarios are listed below:

- If there is no pediatrician at the facility: neonate must be transferred
- If there is a pediatrician at the facility: the neonate can either be treated at the facility or transferred
- If hemodynamic failure or shock occurs: neonate must be transferred

*Sepsis Medical Records from Complete EONC Health Facilities*: Observe the following in the record: neonate was evaluated by a specialist + vital signs checked (pulse + temperature + heart rate + respiratory rate + abdominal examination) + lab tests performed (complete blood count (leukocytes, hemoglobin and hematocrit) + platelet count + oxygen saturation + blood culture + blood glucose test + c-reactive protein + band neutrophils) + antibiotics administered (ampicillin/gentamicin/other antibiotic).

# El Salvador

## El Salvador: Obstetric complications managed according to standards

*Definition:* Women with obstetric complications (sepsis, hemorrhage, severe pre-eclampsia and eclampsia) managed according to the norm in the last two years

*Denominator*:

Total number of obstetric complication records in the sample.

*Formula*:

Sepsis:

*Sepsis Medical Records from Complete EONC Health Facilities*: Vital signs checked (temperature + pulse + diastolic + systolic blood pressure) + leucocyte count + antibiotics administered.

Hemorrhage:

*Hemorrhage Medical Records from Complete EONC Health Facilities*: Vital signs checked (diastolic + systolic blood pressure) + lab tests performed (Ht + Hb + PT + PTT + platelet count) + oxytocin or other uterotonic administered + the cause of hemorrhage recorded.

Severe Pre-Eclampsia / Eclampsia:

*Severe Pre-Eclampsia or Eclampsia Medical Records from Complete EONC Health Facilities*: Vital signs checked (diastolic + systolic blood pressure + pulse + respiratory rate + patellar reflex) + lab tests performed (urine protein +platelet count + aspartate aminotransferase + alanine aminotransferase + lactate dehydrogenase) + outcome of pregnancy recorded + correct treatment given. Correct treatment is evaluated as follows: if diastolic blood pressure is greater than 110 then administration of hydralazine/nifedipine + if gestational age is 26-34 weeks, then administration of dexamethasone/betamethasone + administration of magnesium sulfate.

## El Salvador: Neonatal complications managed according to standards

*Definition:* Neonates with complications (low birth weight, prematurity, birth asphyxia and sepsis) managed according to standards in the last two years

*Denominator*:

Total number of records of neonates with birth complications (low birth weight, prematurity, birth asphyxia, or sepsis) in the sample.

*Formula*:

Low birth weight and prematurity:

*Low Birth Weight Medical Records from Complete EONC Health Facilities*: All checkups reported (pulse + respiratory rate + Downes or Silverman score) + lab tests performed (oxygen saturation level + blood glucose level) + neonate evaluated by a doctor at admission

Asphyxia:

*Asphyxia Medical Records from Complete EONC Health Facilities*: Observe the following in the record: All checkups reported (pulse + respiratory rate + chest radiograph + Downes or Silverman score) + lab tests performed (oxygen saturation level + hemoglobin + C-reactive protein + erythrocyte sedimentation rate + blood glucose level) + treatment with antibiotics.

Sepsis:

*Sepsis Medical Records from Complete EONC Health Facilities*: All checkups reported (temperature + pulse) + lab tests performed (leukocyte count + C-reactive protein + erythrocyte sedimentation rate) + treatment with antibiotics + neonate evaluated by a doctor at admission.

# Guatemala

## Guatemala: Obstetric complications managed according to standards

*Definition:* Women with obstetric complications (sepsis, hemorrhage, severe pre-eclampsia and eclampsia) managed according to the norm in the last two years

*Denominator*:

Total number of obstetric complication records in the sample.

*Formula*:

Sepsis:

*Sepsis Medical Records from Basic EONC Health Facilities*: (temperature + pulse + systolic and diastolic blood pressure) + leukocyte count + antibiotics +referral to Complete EONC

*Sepsis Medical Records from Complete EONC Health Facilities*: (temperature + pulse + systolic BP + diastolic BP) + correct treatment (if septic abortion=manual vacuum aspiration/revision of uterus; if uterine perforation=hysterectomy; if perforations/abscesses /infected ectopic pregnancy=laparotomy; if tears of cervical canal or uterus=surgical repair) + antibiotics administration

Hemorrhage:

*Hemorrhage Medical Records from Basic EONC Health Facilities*: pulse + blood pressure + oxytocin + ringer's lactate + referral to Complete

*Hemorrhage Medical Records from Complete EONC Health Facilities*: Hematocrit + Hemoglobin + PT + PTT + platelet count + diastolic BP + systolic BP + oxytocin/other uterotonics + cause +correct treatment (if complicated abortion/retained placenta=manual vacuum aspiration/revision of uterus; if placenta previa/ placental abruption/ uterine rupture/ uterine atony=C-section / hysterectomy; if ectopic pregnancy/uterine atony=laparotomy; if tears of cervical canal or uterus=surgical repair)

Severe Pre-Eclampsia / Eclampsia:

*Severe Pre-Eclampsia or Eclampsia Medical Records from Basic EONC Health Facilities*: diastolic + diastolic BP + urinary protein + hydralazine/nifedipine (if diastolic BP > 110)

*Severe Pre-Eclampsia or Eclampsia Medical Records from Complete EONC Health Facilities*: systolic BP + diastolic BP + pulse + respiratory rate + patellar reflex +hydralazine / nifedipine (if diastolic BP>110)) + magnesium sulfate + Dexamethasone/ betamethasone (if gestational age 26-34 weeks) + check for urine protein+ platelet count + outcome of pregnancy(C-section/vaginal delivery)

## Guatemala: Neonatal complications managed according to standards

*Definition:* Neonates with complications (low birth weight, prematurity, birth asphyxia and sepsis) managed according to standards in the last two years

*Denominator*:

Total number of records of neonates with birth complications (low birth weight, prematurity, birth asphyxia, or sepsis) in the sample.

*Formula*:

Low birth weight:

*Low Birth Weight Medical Records from Basic EONC Health Facilities*: gestational age + method to calculate gestational age + weight + height + head circumference + skin tone + pulse + respiratory rate + abdominal exam + glycaemia + evaluated by a doctor at admission + oxygen saturation + Silverman or Downs score + referral to Complete EONC

*Low Birth Weight Medical Records from Complete EONC Health Facilities*: pulse + respiratory rate + oxygen saturation + Silverman or Downs score + glycaemia +baby was evaluated by a doctor at admission + (oxygen mask or oxygen hood or oxygen CAAP or mechanical ventilation or keeping in incubator or other) + IV feeding if respiratory rate is >80

Prematurity:

*Prematurity Medical Records from Basic EONC Health Facilities*: gestational age + method of calculating gestational age + weight + height + head circumference + skin exam + pulse + respiratory rate + abdominal exam + glycaemia + exam by doctor at admission + oxygen saturation + Downs or Silverman score + reference to CONE complete

*Prematurity Medical Records from Complete EONC Health Facilities*: pulse + respiratory rate + oxygen saturation + Silverman or Downs score + glycaemia +baby was evaluated by a doctor at admission + oxygen mask/oxygen hood/oxygen CAAP/mechanical ventilation/keeping in incubator/other)+IV feeding if respiratory rate is >80

Asphyxia:

*Asphyxia Medical Records from Basic EONC Health Facilities*: gest age + temp+ skin exam + respiratory rate + pulse + APGAR + glycaemia + evaluated by a doctor at admission

*Asphyxia Medical Records from Complete EONC Health Facilities*: pulse + respiratory rate + radiography + oxygen saturation + Silverman or Downs score + glycaemia +hemoglobin +C-reactive protein +erythrocyte sedimentation rate + chest radiograph +antibiotic treatment +baby was evaluated by a doctor at admission

Sepsis:

*Sepsis Medical Records from Basic EONC Health Facilities*: BP+ temperature + pulse + leukocyte count + C-reactive protein + erythrocyte sedimentation rate + baby was evaluated by a doctor at admission + treatment with antibiotics

*Sepsis Medical Records from Complete EONC Health Facilities*: BP+ temperature + pulse + leukocyte count + C-reactive protein + erythrocyte sedimentation rate + baby was evaluated by a doctor at admission + treatment with antibiotics

# Honduras

## Honduras: Obstetric complications managed according to standards

*Definition:* Women with obstetric complications (sepsis, hemorrhage, severe pre-eclampsia and eclampsia) managed according to the norm in the last two years

*Denominator*:

Total number of obstetric complication records in the sample.

*Formula*:

Sepsis:

*Sepsis Medical Records from Basic EONC Health Facilities*: Observe the following in the record: vital signs checked (temperature + diastolic + systolic blood pressure + pulse) + antibiotics administered + baby referred to another facility.

*Sepsis Medical Records from Complete EONC Health Facilities*: Observe the following in the record: vital signs checked (temperature + diastolic + systolic blood pressure + pulse) + antibiotics administered + correct treatment administered and recorded. Correct treatment is as follows: if septic abortion, then manual aspiration and/or uterine revision recorded + if uterine perforation, then hysterectomy reported + if uterine perforation, abscess, or infected ectopic pregnancy, then laparotomy recorded + if tears of cervical canal or uterus, then surgical repair recorded.

Hemorrhage:

*Hemorrhage Medical Records from Basic EONC Health Facilities*: Observe the following in the record: vital signs checked (diastolic + systolic blood pressure + pulse) + oxytocin or other uterotonic administered + Ringer’s lactate administered + baby referred to another facility.

*Hemorrhage Medical Records from Complete EONC Health Facilities*: Observe the following in the record: vital signs recorded (diastolic + systolic blood pressure) + lab tests performed (Ht + Hb + PT + PTT + platelet count) + oxytocin or other uterotonic administered + a cause of hemorrhage recorded + correct treatment administered and recorded. Correct treatment is as follows: if complicated abortion or retained placenta, then manual aspiration and uterine revision recorded + if placenta previa, placental abruption, uterine rupture, or uterine atony, then Cesarean section or hysterectomy recorded + if ectopic pregnancy or uterine atony, then laparotomy recorded + if tears of the uterine canal or uterus, then surgical repair recorded.

Severe Pre-Eclampsia:

*Severe Pre-Eclampsia Medical Records from Basic EONC Health Facilities*: Observe the following in the record: vital signs checked (diastolic + systolic blood pressure) + urine protein test performed + magnesium sulfate administered + baby referred to another facility.

*Severe Pre-Eclampsia Medical Records from Complete EONC Health Facilities*: Observe the following in the record: vital signs checked (diastolic + systolic blood pressure + pulse + respiratory rate) + lab tests performed (urine protein + platelet count + aspartate aminotransferase + lactate dehydrogenase) + outcome of pregnancy recorded + correct treatment administered and recorded. Correct treatment is as follows: if diastolic blood pressure is greater than 110, then administration of hydralazine/nifedipine recorded + if gestational age is 26-34 weeks, then administration of dexamethasone/betamethasone recorded + administration of magnesium sulfate recorded.

Eclampsia:

*Eclampsia Medical Records from Basic EONC Health Facilities*: Observe the following in the record: vital signs checked (diastolic + systolic blood pressure) + urine protein test performed + magnesium sulfate administered + baby referred to another facility.

*Eclampsia Medical Records from Complete EONC Health Facilities*: Observe the following in the record: vital signs checked (diastolic + systolic blood pressure + pulse + respiratory rate) + lab tests performed (urine protein + platelet count + aspartate aminotransferase + lactate dehydrogenase) + outcome of pregnancy recorded + correct treatment administered and recorded. Correct treatment is as follows: if diastolic blood pressure is greater than 110, then administration of hydralazine/nifedipine recorded + if gestational age is 26-34 weeks, then administration of dexamethasone/betamethasone recorded + administration of magnesium sulfate recorded.

## Honduras: Neonatal complications managed according to standards

*Definition:* Neonates with complications (low birth weight, prematurity, birth asphyxia and sepsis) managed according to standards in the last two years

*Denominator*:

Total number of records of neonates with birth complications (low birth weight, prematurity, birth asphyxia, or sepsis) in the sample.

*Formula*:

Low birth weight and prematurity:

*Low Birth Weight and Prematurity Medical Records from Complete EONC Health Facilities*: Observe the following in the record: vital signs checked (weight + respiratory rate + blood pressure + Silverman score) + oxygen saturation level + blood glucose level checked + baby evaluated by a doctor + correct treatment administered and recorded. Correct treatment is as follows: oxygen mask/oxygen hood/oxygen tank/mechanical ventilation/keeping in incubator recorded + IV feeding recorded if respiratory rate greater than 80.

Asphyxia:

*Asphyxia Medical Records from Complete EONC Health Facilities*: Observe the following in the record: vital signs checked (respiratory rate + Silverman score) + checks/lab tests performed (oxygen saturation level + Hb + blood glucose level) + correct treatment administered and recorded. Correct treatment is as follows: oxygen mask/oxygen hood/oxygen tank/mechanical ventilation/keeping in incubator recorded.

Sepsis:

*Sepsis Medical Records from Complete EONC Health Facilities*: Observe the following in the record: vital signs checked (temperature + blood pressure) + lab tests performed (oxygen saturation level checked + leukocyte count) + antibiotics administered + baby evaluated by a doctor.

# Chiapas, Mexico

## Chiapas: Obstetric complications managed according to standards

*Definition:* Women with obstetric complications (sepsis, hemorrhage, severe pre-eclampsia and eclampsia) managed according to the norm in the last two years

*Denominator*:

Total number of obstetric complication records in the sample.

*Formula*:

Sepsis:

*Sepsis Medical Records from Basic EONC Health Facilities*: Observe the following in the record: vital signs checked (systolic blood pressure + diastolic blood pressure + temperature + pulse) + lab tests performed (leukocyte count) + antibiotic administered (amikacin, clindamycin, gentamicin, ampicillin, metronidazole, or other antibiotic) + transferred to another facility.

*Sepsis Medical Records from Complete EONC Health Facilities*: Observe the following in the record: vital signs checked (systolic blood pressure + diastolic blood pressure + temperature + pulse) + medication administered (amikacin, clindamycin, gentamicin, ampicillin, metronidazole, or other antibiotic).

Hemorrhage:

*Hemorrhage Medical Records from Basic EONC Health Facilities*: Observe the following in the record: vital signs checked (pulse + diastolic blood pressure + systolic blood pressure) + medication administered (oxytocin/other uterotonic + Ringer’s lactate) + result was recorded.

*Hemorrhage Medical Records from Complete EONC Health Facilities*: Observe the following in the record: vital signs checked (diastolic blood pressure + systolic blood pressure) + lab tests performed (Ht + Hb + PT + PTT + platelet count) + medication administered (oxytocin/other uterotonic) + cause of hemorrhage recorded.

Severe Pre-Eclampsia:

*Severe Pre-Eclampsia Medical Records from Basic EONC Health Facilities*: Observe the following in the record: vital signs checked (systolic blood pressure + diastolic blood pressure) + lab tests performed (urine protein) + medication administered (magnesium sulfate + hydralazine/nifedipine/other hypertensive (if diastolic blood pressure is >110)).

*Severe Pre-Eclampsia Medical Records from Complete EONC Health Facilities*: Observe the following in the record: vital signs check (systolic blood pressure + diastolic blood pressure + pulse + respiratory rate) + lab tests performed (urine protein + platelet count + aspartate aminotransferase + alanine aminotransferase + lactate dehydrogenase) + medication administered (magnesium sulfate + hydralazine/nifedipine/other hypertensive (if diastolic blood pressure is >110) + dexamethasone/betamethasone (if gestational age is 26-34 weeks)) + outcome of pregnancy recorded.

Eclampsia:

*Eclampsia Medical Records from Basic EONC Health Facilities*: Observe the following in the record: vital signs checked (systolic blood pressure + diastolic blood pressure) + lab tests performed (urine protein) + medication administered (magnesium sulfate + hydralazine/nifedipine/other hypertensive (if diastolic blood pressure is >110)).

*Eclampsia Medical Records from Complete EONC Health Facilities*: Observe the following in the record: vital signs check (systolic blood pressure + diastolic blood pressure + pulse + respiratory rate) + lab tests performed (urine protein + platelet count + aspartate aminotransferase + alanine aminotransferase + lactate dehydrogenase) + medication administered (magnesium sulfate + hydralazine/nifedipine/other hypertensive (if diastolic blood pressure is >110) + dexamethasone/betamethasone (if gestational age is 26-34 weeks)) + outcome of pregnancy recorded.

## Chiapas: Neonatal complications managed according to standards

*Definition:* Neonates with complications (low birth weight, prematurity, birth asphyxia and sepsis) managed according to standards in the last two years

*Denominator*:

Total number of records of neonates with birth complications (low birth weight, prematurity, birth asphyxia, or sepsis) in the sample.

*Formula*:

Low birth weight:

*Low Birth Weight Medical Records from Basic EONC Health Facilities*: Observe the following in the record: neonate was evaluated by a doctor + gestational age + method used to calculate gestational age + vital signs checked (weight + height + head circumference + skin color + pulse + respiratory rate + abdominal examination + Silverman score) + lab tests performed (blood glucose level + oxygen saturation level) + transferred/referred to a complete facility.

*Low Birth Weight Medical Records from Complete EONC Health Facilities*: Observe the following in the record: neonate was evaluated by a doctor + vital signs checked (pulse + respiratory rate + Silverman score) + lab tests performed (blood glucose level + oxygen saturation level).

Prematurity:

*Prematurity Medical Records from Basic EONC Health Facilities*: Observe the following in the record: neonate was evaluated by a doctor + gestational age + method used to calculate gestational age + vital signs checked (weight + height + head circumference + skin color + pulse + respiratory rate + abdominal examination + Silverman score) + lab tests performed (blood glucose level + oxygen saturation level) + transferred/referred to a complete facility.

*Prematurity Medical Records from Complete EONC Health Facilities*: Observe the following in the record: neonate was evaluated by a doctor + vital signs checked (pulse + respiratory rate + Silverman score) + lab tests performed (blood glucose level + oxygen saturation level).

Asphyxia:

*Asphyxia Medical Records from Basic EONC Health Facilities*: Observe the following in the record: Observe the following in the record: neonate was evaluated by a doctor + gestational age + vital signs checked (temperature + skin color + pulse + respiratory rate + abdominal examination + Apgar score (at 1 or 5 minutes)) + lab tests performed (blood glucose level) + treatment with antibiotics (ampicillin/gentamicin/other antibiotic).

*Asphyxia Medical Records from Complete EONC Health Facilities*: Observe the following in the record: vital signs checked (pulse + respiratory rate + Silverman score) + lab tests performed (oxygen saturation level + blood glucose level + hemoglobin + c-reactive protein + erythrocyte sedimentation rate + chest x-ray) + treatment with antibiotics (ampicillin/gentamicin/other antibiotic).

Sepsis:

*Sepsis Medical Records from Basic EONC Health Facilities*: Observe the following in the record: neonate was evaluated by a doctor + gestational age + vital signs checked (temperature + skin color + pulse + respiratory rate + abdominal examination) + lab tests performed (leukocyte count + neutrophil morphology + platelet count + blood glucose level) + treatment with antibiotics (ampicillin/gentamicin/other antibiotic) + transferred/referred to a complete facility.

*Sepsis Medical Records from Complete EONC Health Facilities*: Observe the following in the record: neonate was evaluated by a doctor + vital signs checked (temperature + pulse) + lab tests performed (leukocyte count + c-reactive protein + erythrocyte sedimentation rate) + treatment with antibiotics (ampicillin/gentamicin/other antibiotic).
